# Effects of Cetuximab and Erlotinib on the behaviour of cancer stem cells in head and neck squamous cell carcinoma

**DOI:** 10.18632/oncotarget.24416

**Published:** 2018-02-05

**Authors:** Maria Fernanda Setúbal Destro Rodrigues, Luke Gammon, Muhammad M. Rahman, Adrian Biddle, Fabio Daumas Nunes, Ian C. Mackenzie

**Affiliations:** ^1^ Oral Pathology Department, School of Dentistry, University of São Paulo, São Paulo, Brazil; ^2^ Blizard Institute, Barts and the London School of Medicine and Dentistry, Queen Mary University of London, London, UK

**Keywords:** HNSCC, cancer stem cells, Cetuximab, EMT, differentiation

## Abstract

The therapeutic responses of many solid tumours to chemo- and radio-therapies are far from fully effective but therapies targeting malignancy-related cellular changes show promise for further control. In head and neck squamous cell carcinoma, the epidermal growth factor receptor (EGFR) is commonly overexpressed and investigation of agents that block this receptor indicate a limited response when used alone but an ability to enhance the actions of other drugs. The hierarchical stem cell patterns present in tumours generate cellular heterogeneity and this is further complicated by cancer stem cells (CSC) shifting between epithelial (Epi-CSC) and mesenchymal (EMT-CSC) states. To clarify how such heterogeneity influences responses to EGFR blocking, we examined the effects of Cetuximab and Erlotinib on the cell sub-populations in HNSCC cell lines. These agents reduced cell proliferation for all subpopulations but induced little cell death. They did however induce large shifts of cells between the EMT-CSC, Epi-CSC and differentiating cell compartments. Loss of EMT-CSCs reduced cell motility and is expected to reduce invasion and metastasis. EGFR blocking also induced shifts of Epi-CSCs into the differentiating cell compartment which typically has greater sensitivity to chemo/radiation, an effect expected to enhance the overall response of tumour cell populations to adjunctive therapies.

## INTRODUCTION

Head and neck cancers are among the 10 most common cancers worldwide and the great majority of these cancers are squamous cell carcinomas and associated with severe mortality [1–3]. Currently the usual treatment for HNSCC patients remains surgery in combination with radiotherapy and this is further combined with chemotherapy for more advanced disease and for recurrent and metastatic tumours [4–6]. Despite therapeutic advances during the last decade, the 5-year-survival rate for HNSCC remains low with late diagnosis at advanced tumour stages typically associated with local and regional recurrences, and with development of lymph node and distant metastasis [7, 8]. The poor therapeutic responses of HNSCC have encouraged focused efforts towards development of molecular targeted therapies [7, 9, 10].

Overexpression of the epidermal growth factor receptor (EGFR) is a frequent molecular alteration in HNSCC and increased activity of the EGF pathway has been associated with resistance to treatment and poor clinical outcome [5, 11, 12]. The EGFR is a member of the HER tyrosine kinase receptor family and binding of specific ligands, such as epidermal growth factor (EGF) and transforming growth factor alpha (TGF-α), promotes homo- or hetero-dimerization of EGFR family members and activation of intracellular signaling pathways that control growth, differentiation, survival and invasion [13–15]. The EGFR is therapeutically targeted by agents such as Cetuximab and Erlotinib [6] but the modest effects observed on tumour control have been rather disappointing considering the importance of EGFR-initiated signaling pathways. Various mechanisms for primary and acquired resistance to Cetuximab have been suggested and include constitutive activation of EGFR-mediating signaling molecules and activation of alternative ErbB2 and ErbB3 pathways [16, 17].

There is now substantial evidence that HNSCC, like other solid tumours, consist of a heterogeneous population of cells. This results partly from the evolution of clonal heterogeneity [18] but is also due to persistence of a hierarchy of stem and differentiating cells similar to that present in normal tissues [19, 20]. Cancer stem cells (CSCs) are identified as a subpopulation of cells with the ability to self-renew indefinitely whilst also generating daughter cells that differentiate, usually aberrantly, and the self-renewal properties of CSCs are tested by their selective ability to initiate tumours in immune-deficient mice [21]. The CSC subpopulation not only drives tumour growth but is also responsible for the aggressiveness and therapeutic resistance of tumours [22]. CSCs in HNSCC were initially identified and isolated by their high levels of expression of the hyaluronan receptor CD44 [19, 23, 24] and several subsequent reports have described isolation of CSCs from HNSCC using either CD44 or various other markers [25]. CSCs also persist in malignant cell lines and can be identified by high levels of CD44 expression, by morphological characteristics, and by their clonogenicity [23].

Of particular interest, tumour invasion and metastasis have been linked to epithelial-mesenchymal transition (EMT), a process during which cells lose epithelial features of mutual attachment and gain mesenchymal features, including mobility [26–29]. It is proposed that EMT enables CSCs to invade surrounding local and distant tissues where the reverse process of mesenchymal-to-epithelial transition (MET) enables their return to the epithelial phenotype to generate metastatic growth [30]. Biddle and co-workers [31] demonstrated that the self-renewing CD44^high^ CSC fraction of HNSCC contains as at least two distinct cellular phenotypes with marked differences in cell motility, morphology, proliferation, clonogenicity, and sphere forming abilities. Both cell fractions are tumour-initiating and both express high levels of CD44 but the CSCs with an epithelial phenotype express high levels of ESA whereas those that have undergone EMT have low or absent levels of ESA [31]. CSCs have the ability to switch between these phenotypes and, as a result, aggressive tumours consist of at least 3 cell types, epithelial CSCs, mesenchymal CSCs, and a differentiating non-stem cell fraction [31]. A comprehensive understanding of drug actions will therefore depend on information about the individual responses of each of these cell types.

Apart from their EMT-mediated roles in invasion and metastasis, the therapeutic importance of CSCs lies in their greater ability to resist therapeutic killing in response to radio- and chemo-therapies [22, 32, 33]. Differential effects of CSC sub-fractions to the molecularly targeted therapies of agents such as Cetuximab and Erlotinib are therefore of interest [34]. We have now investigated the effects of blocking EGFR function on each of the phenotypically-different cell sub-populations in HNSCC cell lines and find that EGFR inhibition leads to morphological and molecular changes, marked reductions in cell proliferation, but no marked induction of cell death. It leads to reduced CD44 expression and a shift of CSCs into differentiation, a state in which they have enhanced therapeutic sensitivities. This action provides a form of “differentiation therapy” [35–37] and appears related to the greater effectiveness of EGFR inhibition when used as an adjunctive component of therapies. Both Cetuximab and Erlotininib also shift EMT-CSCs back into the epithelial phenotype, a change that may tend to restrict local invasion and metastasis.

## RESULTS

### Effects of Cetuximab and Erlotinib on cell proliferation and apoptosis in HNSCC cell lines.

**Figure 1:**
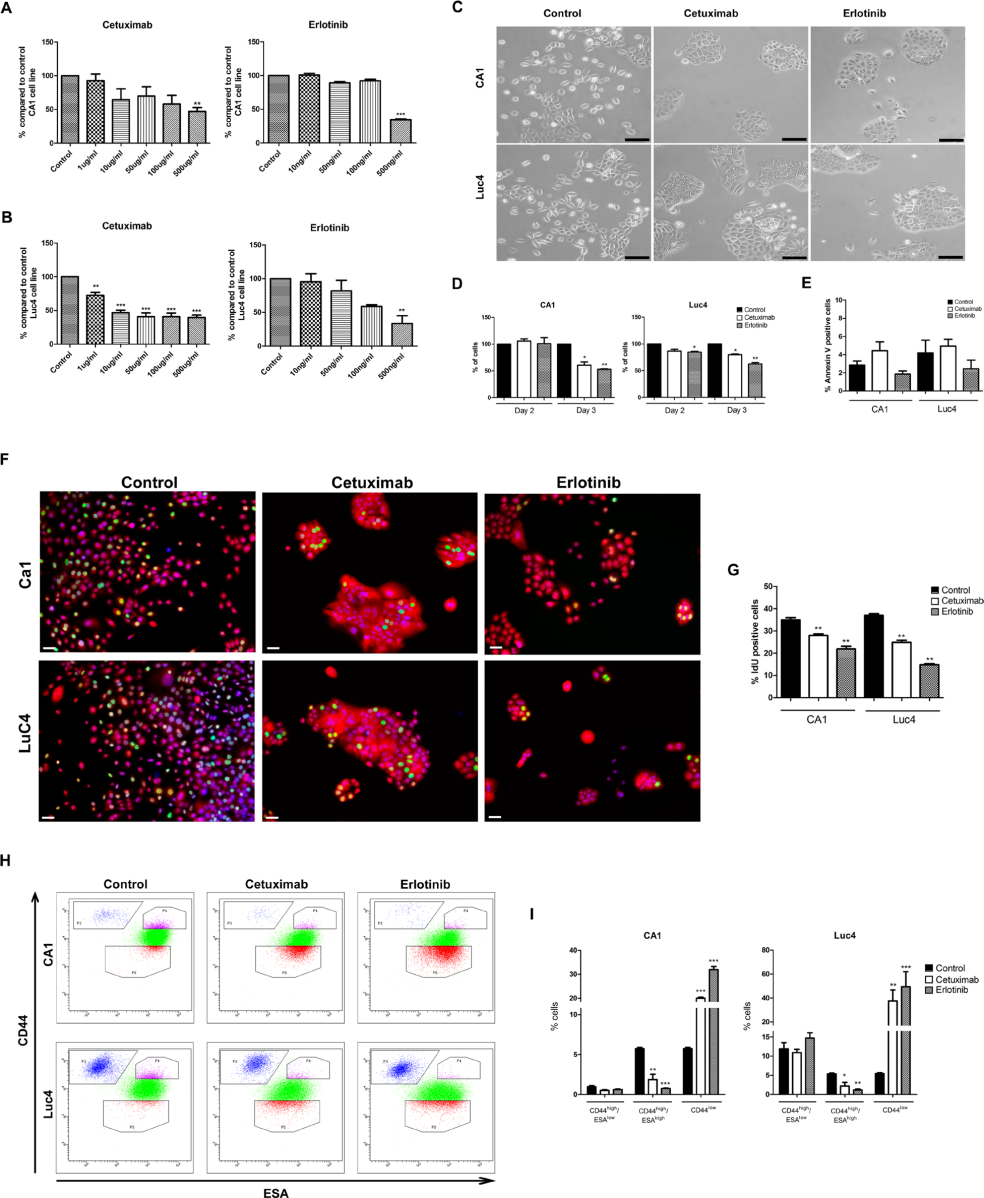
Effects of Cetuximab and Erlotinib on cell proliferation and apoptosis in HNSCC cell lines Percentage of total number of CA1 (**A**) and Luc4 (**B**) cells compared to controls after 3 days of treatment with Cetuximab and Erlotinib at various concentrations. (**C**) Cell morphology of CA1 and Luc4 after treatment with Cetuximab (100 µg/ml) and Erlotinib (500 ng/ml) for 3 days (Scale = 50μm). (**D**) Total number of CA1 and Luc4 after each day of treatment. (**E**) Analysis of Annexin V positive cells after 3 days treatment with either Cetuximab or Erlotinib. (**F**) Representative images of IdU positive cells (green) in CA1 and Luc4 after treatment with Cetuximab and Erlotinib (Scale = 25μm). (**G**) Quantification of the number of IdU positive cells. (**H**) FACS analysis of expression of CD44 and ESA on CA1 and Luc4 cells after 3 days of treatment with Cetuximab or Erlotinib showing decrease of cells with CD44^high^/ESA^high^ expression and increase the CD44^high^/ESA^low^ fraction. (**I**) Quantification of data depicted in H, showing the percentage of CD44^high^/ESA^low^, CD44^high^/ESA^high^ and CD44^low^ fractions. For this and all subsequent figures; ^*^P < 0.05, ^**^P < 0.01, ^***^P < 0.001.

Treatment levels were initially determined by treating CA1 and Luc4 cell lines for 3 days with drug concentrations of Cetuximab and Erlotinib ranging from 0–500 µg/ml or 0–500 ng/ml respectively (Figure 1A, 1B). Based on these pilot data, Cetuximab at a dose of 100 µg/ml, and Erlotinib at a dose of 500 ng/ml, were used for subsequent experiments. By day 3, both drugs induced marked morphological changes, including the formation of more compact cell colonies and loss of individual elongated EMT-like cells (Figure 1C). Cell cultures were examined to assess the extent to which the reduced cell numbers present after 3 days of treatment (Figure 1D) were related to reduced proliferation or to increased apoptosis. Cytometry after staining for Annexin V and DAPI showed low (2–4%) levels of apoptosis in untreated cells. The lack of significant increases in counts of Annexin V positive cells after treatment indicated no marked promotion of apoptotic cell death (Figure 1E). Effects of treatment in markedly reducing rates of cell cycling were indicated by significant decreases in IdU labeled cells (Figure 1F, 1G).

### Cetuximab and Erlotinib decrease the levels of CD44 expression of CA1 and Luc4 cells.

To further assess the effects of EGFR inhibitors, we examined whether there were differential changes in the cell line sub-fractions we have previously identified . Cells were stained for CD44 and ESA and fractionated by flow cytometry into CD44^high^/ESA^low^ (EMT-CSC), CD44^high^/ESA^high^ (EPI-CSC) and CD44^low^ (NON-CSC) populations. For both cell lines, treatment significantly decreased the percentage of CD44^high^/ESA^high^ cells and consistently increased the percentage of CD44^low^ cells (Figure 1H, 1I). Following treatment CD44^high^/ESA^low^ fractions showed no statistically significant differences in mean CD44 or ESA expression levels, and the proportion of this fraction, expressed as a percentage of the total cells, also remained unchanged.

**Figure 2:**
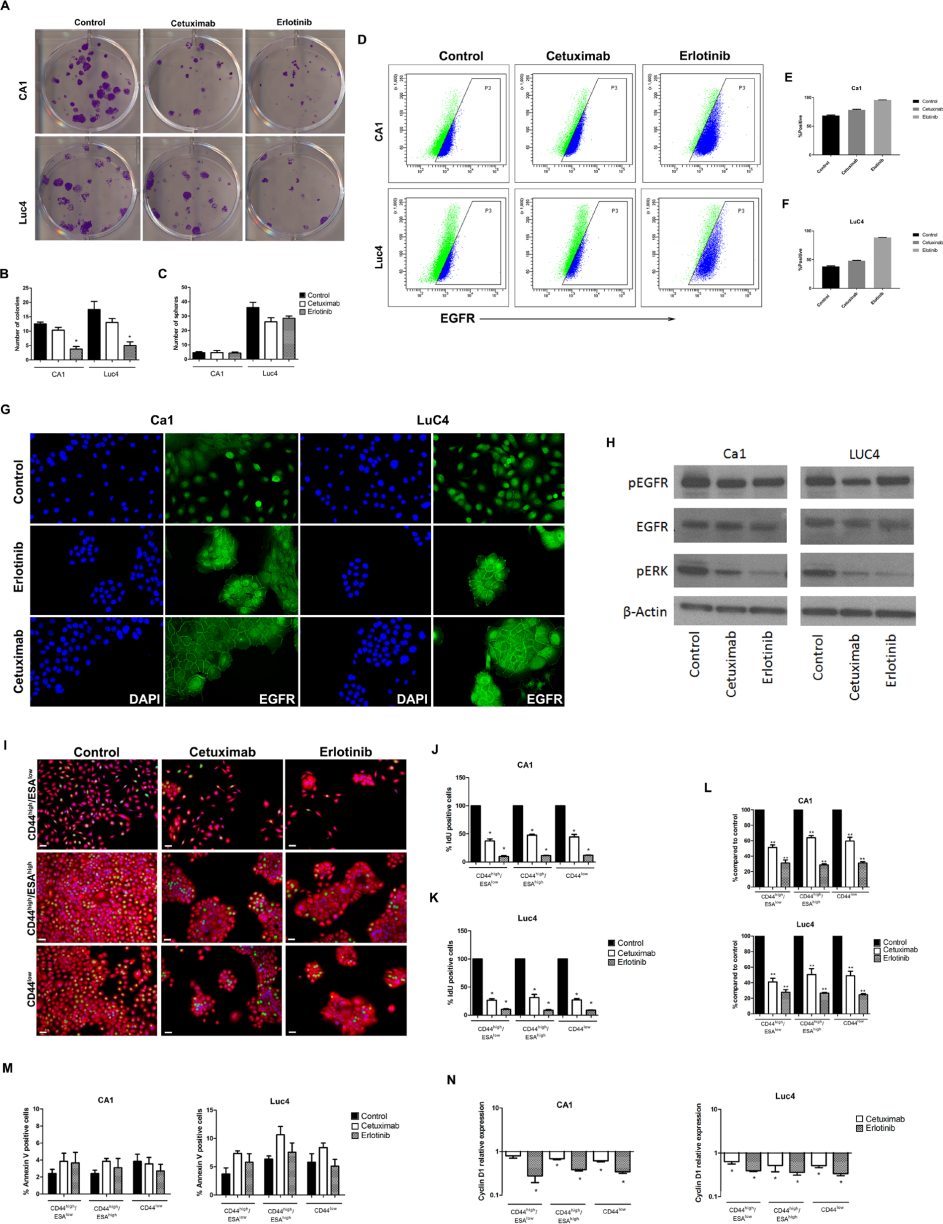
Cetuximab and Erlotinib decrease clonogenicity, proliferation rates and EGFR expression (**A**) Effects of treatment on colony forming ability. (**B**) Quantification of the number of colonies formed in CA1 and Luc4 cell lines. (**C**) Number of spheres formed after 3 days of treatment. (**D**) FACS plots of changes in the number of cells expressing cell-surface EGFR. (**E**, **F**) Comparisons of percentages of EGFR expressing cells in the two cell lines after treatment. (**G**) Altered patterns of EGFR staining after treatment. (**H**) Western blots showing protein levels for EGFR, pEGFR, and pERK. Altered patterns of cell proliferation (**I**–**K**), accumulation (**L**) and apoptosis (**M**) of cell sub-fractions following treatment. (**N**) Levels of Cyclin D1 were reduced in all sub-fractions.

To determine whether the decreases in CD44 expression indicated by flow cytometry were associated with functional decreases in stemness, treated cell lines were re-plated at low density to assess their colony-forming abilities. Treated cells of both cell lines showed significant reductions in colony forming ability (Figure 2A and 2B) but no significant differences in their ability to form tumour spheres (Figure 2C).

### Cetuximab and Erlotinib alter EGFR expression patterns

Differences in EGFR expression induced by Cetuximab and Erlotinib were evaluated by flow cytometry and western blotting. Plots of EGFR versus side-scatter indicated that the control populations of both cell lines had similar and substantial levels of total cell surface EGFR and that these levels increased following treatment (Figure 2D–2F). Control populations of both CA1 and Luc4 showed higher expression of EGFR on the CD44^high^/ESA^high^ Epi-CSC subfraction than on either CD44^high^/ESA^low^ or CD44^low^ cells and there was a trend for expression in all fractions to increase after treatment. Immunofluorescence showed cytoplasmic staining with higher than control levels of EGFR at the cell peripheries of the cohesive cell colonies formed following treatment (Figure 2G). Following treatment, Western blots indicated little difference in the overall levels of pEGFR or EGFR but showed reduced levels of the downstream target pERK indicated that both inhibitors functioned to interrupt the primary EGFR signaling pathway (Figure 2H).

### Cetuximab and Erlotinib decrease the proliferation rates of all cell fractions and prevent G1/S progression

To evaluate proliferative effects of Cetuximab or Erlotinib treatment on the CD44^high^/ESA^low^, CD44^high^/ESA^high^ and CD44^low^ fractions, cells were sorted and re-plated. Treatment resulted in a reduced accumulation of cells for each of the sorted fractions of both cell lines with levels of IdU incorporation significantly and similarly reduced for all sub-fractions (Figure 2I–2L). Counts of Annexin V positive cells indicated low levels of apoptosis in control specimens and treatment-induced changes in apoptosis were small and not significant for any of the 3 cell sub-fractions (Figure 2M). Cyclin D1, which is required for progression of cells through the G1/S cell cycle phase, also showed significant decreases in all cell fractions with both treatments (Figure 2N).

### Cetuximab and Erlotinib increase cell differentiation

**Figure 3:**
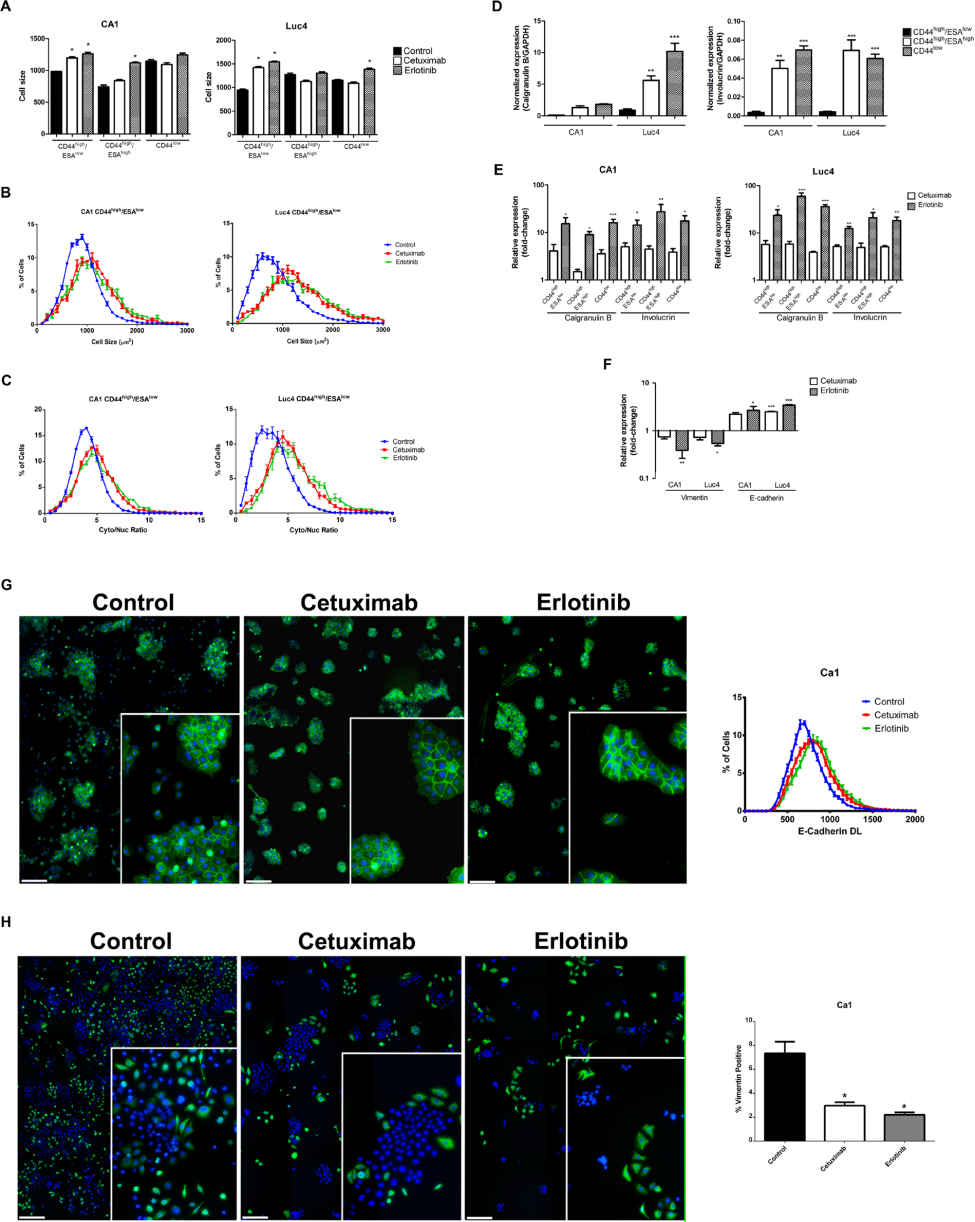
Cetuximab and Erlotinib induce cellular differentiation (**A**) Mean cell size of CD44^high^/ESA^low^, CD44^high^/ESA^high^ and CD44^low^ fractions in CA1 and Luc4 cell lines after treatment. Representative graphs showing (**B**) increased size and (**C**) increased cytoplasmic/nuclear ratio of CD44^high^/ESA^low^ cells. In control cultures, expression of the differentiation markers Calgranulin B and Involucrin is normally lowest in CD44^high^/ESA^low^ sub-population (**D**) but is increased in all fractions (**E**) after treatment. (**F**) qPCR indicated reduced Vimentin and increased E-cadherin expression of EMT fractions after treatment. (**G**) Control cultures show some cohesive colonies staining for E-cadherin (green) surrounded by scattered DAPI-positive E-cadherin negative cells. After treatment with Cetuximab or Erlotinib scattered cells were lost and nearly all cells were E-cadherin positive. (**H**) Control cultures show numerous scattered vimentin-positive (green) cells which, after treatment are reduced in number and tend to cluster into and around epithelial-like colonies (Scale for G and H = 25 μm) Inserts show colonies at higher magnification.

The observed treatment effects of loss of cells from the CD44^high^/ESA^high^ cell fractions, reduction in colony forming assays, and an increased proportion of CD44^low^ cells indicated a shift of cells from the Epi-CSCs stem cell compartment into differentiation. To assess other indicators of differentiation, we examined changes in cell size and in cytoplasmic-to-nuclear ratio as these are known to increase with differentiation. Following treatment, sorted cell fractions showed trends towards an increased cell size, mean cell area and cytoplasmic to nuclear ratio (Figure 3A–3C). Expression of the epithelial differentiation markers Calgranulin B and Involucrin was found to be lowest in the CD44^high^/ESA^low^ EMT cell fractions, and the highest in the CD44^low^ fractions and significant increases in these markers for all cell fractions indicated that treatment resulted in shifts towards differentiation (Figure 3D, 3E).

### Cetuximab and Erlotinib effects on the size of EMT cell fractions, rates of cell migration, and cisplatin responses

Control cultures showed populations of scattered cells with an EMT-like appearance and their loss after treatment suggesting a transition of EMT cells back to an epithelial-like phenotype. As little increase in ESA expression was detected by FACS, we examined FACS-isolated CD44^high^/ESA^low^ EMT fractions for other evidence of mesenchymal-epithelial transition (MET). QPCR (Figure 3F) and immunofluorescence staining cell indicated significant decreases in vimentin expression and increases in E-cadherin expression in treated populations (Figure 3F–3H). The loss of scattered e-cadherin negative/vimentin positive cells seen in control cultures was again associated with cell clustering to form compact colonies.

**Figure 4:**
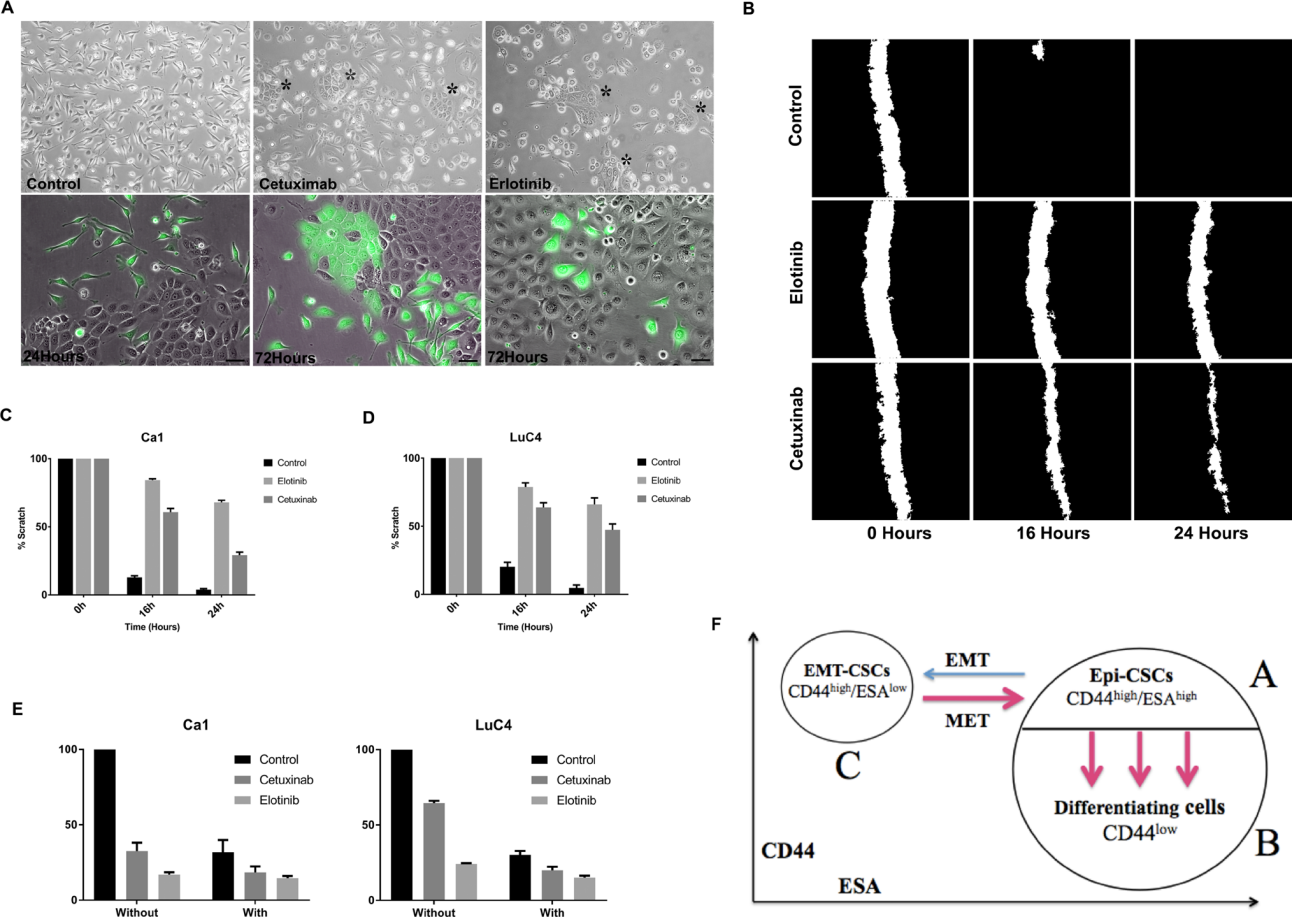
Changes in cell transitions, motility, and response to cisplatin induced by treatment (**A**) Isolated populations of CD44^high^/ESA^low^ cells retain EMT characteristics 3 days after plating but treated with Cetuximab or Erlotinib begin to form clustered epithelial colonies (^*^). Three days after plating a mixture of EGFP^**+**^ EMT cells with unlabeled parental cells, EGFP^**+**^ cells retain their EMT-like morphology but treatment with Cetuximab or Erlotinib induces transition into an epithelial phenotype (Scale = 25μm). (**B**–**D**). Images of scratches made in control and treated cultures showed reduced closure of scratches after treatment. (**E**) Cell counts after treatment with Cisplatin alone or combined with EGFR inhibition indicate more effective action of the combined drugs. (**F**) Cytometry assessing CD44 and ESA expression identifies 3 cell sub-populations in HNSCC cell lines. The majority cell population is ESA^high^ and contains a CD44^high^/ ESA^high^ epithelial stem cell population (A), and a CD44^low^ differentiating non-stem cell population (B) (see Figure 2A). A third population (C) is CD44^high^/ESA^low^ and corresponds to cells that have undergone EMT. Three transitions occur – EMT, MET and transition into differentiation. Blocking the EGFR markedly enhanced the transitions indicated by red arrows.

A shift of EMT cells towards an epithelial phenotype was assessed in two other ways. When FACS-isolated EMT-CSCs were plated and grown for 3 days, cells from control cultures maintained an EMT-like appearance whereas treated cultures began to transit into cell clusters with an epithelial appearance (Figure 4A, top). The contribution of EMT-CSCs to the compact colonies was apparent when EMT-CSCs were isolated from EGFP-labeled cultures and mixed and plated with unlabeled CD44^high^/ESA^high^ EPI-CSCs. These cultures initially showed individual elongated EGFP-labeled cells that were distinct from the unlabeled epithelial colonies but by 72 hours after treatment clusters of EGFP-labeled cells were present within and around the epithelial colonies (Figure 4A, lower) indicating a morphological transition from EMT-CSCs into an epithelial state. Scratch assays showed marked changes in cell motility as a result of EGFR blocking. Scratches in control wells were essentially closed within 16 hours whereas after EGFR inhibition scratches remained open even at 24 hours (Figure 4B–4D). Cultures treated with Cisplatin were treated with Cetuximab or Erlotinib 3 days later to see whether EGFR blocking enhanced the effects of Cisplatin treatment. Both cell lines showed the enhanced effects on the reduction in cell number when the drugs were used in combination (Figure 4E).

## DISCUSSION

Activation of the EGFR has widespread effects on cell proliferation, survival, invasion, DNA repair and drug resistance. This receptor is frequently overexpressed in HNSCC and has been extensively studied as a molecular target [38]. However, blocking EGFR signaling has provided less therapeutic benefit than initially anticipated [39] and this may be largely related to the cellular heterogeneity of tumours and, particularly, to the presence of sub-populations of CSCs and differentiating cells [40]. There is now considerable evidence that elimination of CSCs is required for successful therapy [26, 41] but it has also been found that CSCs generally have greater resistance to therapeutic killing by either radiation or chemotherapy [32, 42]. Therapeutic manipulation of CSCs is further complicated by their presence as heterogeneous populations of epithelial and mesenchymal phenotypes that differ in their therapeutic responses to individual therapies and are able to switch from one phenotype to the other [31, 40]. Information about the particular specificities of individual drugs for each type of cell may therefore be helpful in developing combinations of drugs that provide effective actions upon the total tumour population, potentially also at lower therapeutic concentrations.

CD44 has been widely used as a general marker for CSC identification [23, 25] and has functions related to stem cell maintenance. For example, CD44 knockout mice show reduced epidermal differentiation [43] and CD44 down-regulation in tumour cells is associated with stem cell loss whereas over-expression of CD44 drives tumour progression and promotes CSC properties [44–46]. EGFR activation increases CD44 expression, blocking EGFR tyrosine kinase domains reduces both stem cell maintenance and EMT, and loss of CD44 down-regulates both total and phosphorylated EGFR [46, 47]. Taken together, these findings support the concept that drugs blocking EGFR signalling are likely to affect CSC maintenance, either directly or indirectly, and that information about differential effects of drugs on stem cell fractions is of therapeutic interest.

Cetuximab and Erlotinib interrupt EGFR signalling by different mechanisms but both strongly inhibited cell proliferation and produced distinct and similar morphological changes. Flow cytometry analysis indicated interesting drug effects on the CSC and non-CSC subpopulations in terms of shifts of cells between these sub-fractions. The proportion of cells in CD44^high^/ESA^high^ Epi-CSC fractions was markedly reduced and the proportion of cells in CD44^low^ fractions was correspondingly raised. The rates of cell proliferation and apoptosis for these subpopulations were not differentially affected by treatment with either drug. Therefore, the reduction in CD44^high^/ESA^high^ Epi-CSCs was attributed to their enhanced transit into the differentiating CD44^low^ population. We have previously shown that clonogenic ability, assessed as the formation of adherent colonies, is primarily a property of Epi-CSCs [31] and the markedly reduced colony-forming efficiency of treated cells supports the concept of loss of Epi-CSCs by differentiation. Further evidence for this shift was found in the increased size of cells in this fraction and their increased expression of the Involucrin and Calgranulin B differentiation markers.

Treated cultures showed a striking disappearance of the numerous scattered cells in control cultures that have an EMT-like appearance. Given this apparent loss of EMT cells by morphological criteria, it was surprising that EGFR inhibition induced no statistically significant differences in the number of cells expressing the CD44^high^/ESA^low^ cell surface phenotype, typically used as an identifier of EMT-CSCs in FACS assay [48]. Nor was there a change in sphere forming ability. Sphere formation has been used to assess the size of “functional” EMT-CSC fractions [31] and the Luc4 line, which has a larger fraction of CD44^high^/ESA^low^ EMT-CSCs than the CA1 cell line, accordingly showed more sphere formation. However, although drug treatment did not induce consistent changes in sphere formation, the decreased Vimentin and increased E-cadherin levels found for sorted CD44^high^/ESA^low^ fractions indicated that some degree of transition of EMT-CSCs towards MET had occurred. This was also demonstrated by the treatment-induced shifts of EGFP-labelled EMT cells into epithelial colonies. The treatment-induced EMT changes found therefore differed depending on the assays employed. However, ascribing a simple binary transition to epithelial and mesenchymal cells undergoing EMT/MET processes is perhaps over simplistic [11]. Stable hybrid epithelial/mesenchymal phenotypes that co-express both epithelial and mesenchymal markers are described [31, 49–51] and the EMT-CSC subpopulation itself appears to be heterogeneous with expression of markers such as Aldehyde dehydrogenase 1 marking cells that retain lineage plasticity [31]. The lack of re-expression of ESA and shifts in sphere formation after EGFR blocking may represent partial transition within a hybrid epithelial/EMT phenotype. Alternatively, a 3-day period of treatment may be insufficient for the full transition of all properties.

The presence of cells expressing mesenchymal markers has previously been reported in breast, prostate and other carcinoma cell lines [11, 52, 53]. In HNSCC cell lines, EMT generates cells with a CD44^high^/ESA^low^ phenotype that are plastic, able to revert to the epithelial phenotype, and relatively resistant to radiation and various chemotherapeutic agents [11, 32, 48]. Mesenchymal cells in HNSCC have been variously identified by a CD44^high^/EGFR^low^ cell surface expression pattern [34], by a Vimentin^high^/E-cadherin^low^ staining pattern [54] and by a CD44^high^/Aldh^high^ staining pattern [55]. Mesenchymal cells are also found in epidermoid and oesophageal carcinoma cell lines [56] and it appears that EMT and MET cell transitions are a common feature of carcinomas [37]. An interesting interpretation of the EMT process is that it “generates cells with the properties of stem cells” [29, 57], a conclusion based mainly on the EMT-induced shift from a CD44^high^/CD24^high^ to a CD44^high^/CD24^low^ phenotype in mammary cell lines [57]). Although the latter corresponds to the phenotype of tumour initiating stem cells in breast cancer [58], other evidence shows that the shift of phenotypic properties induced by EMT occurs within the stem cell compartment itself, i.e. between CD44^high^/CD24^high^ Epi-CSCs and CD44^high^/CD24^low^ EMT-CSCs [31, 48]. As both Epi-CSCs and EMT-CSCs are self-renewing and capable of initiating and maintaining tumours, both cell types need to be eliminated for successful therapy [41, 59].

In response to environmental stimuli, Epi-CSCs transit into self-renewing CD44^high^/ESA^low^ EMT cells and can also revert back to the Epi-CSC phenotype by MET. For a given cell line, balanced rates of the EMT and MET transitions result in a relatively stable size of the EMT-CSC fraction. Typically the rate of these transitions is slow taking, for example, 3 weeks for levels of EMT cells to be greatly increased by hypoxia or by TGFβ, and taking a similar time after withdrawal of stimuli for EMT cells to return to baseline levels [34, 60]. EGFR blocking greatly increased the rate of MET and within 3 days there was loss of cells with an EMT-like appearance and transition of labeled EMT-CSCs back into epithelial colonies. The expected effect of a rapid shift of cells out of the EMT fraction is to reduce the invasive and metastatic potential of the tumour population.

Many reports indicate that the clinical response rate for patients with advanced HNSCC is low when treated with Cetuximab alone, but survival and loco-regional control are improved when patients are treated with Cetuximab in combination with radiotherapy, platinum-based therapy or fluorouracil [5, 12, 51, 61]. Similarly, patients with recurrent or metastatic HNSCC show only a 4.3% response rate to Erlotinib as a monotherapy [62], but have a response rate of 70% for Erlotinib combined with cisplatin or radiotherapy [63, 64]. The present findings of flux between epithelial compartments, each having differing drug responses, may help to explain these observations. EGF has roles in both in initiating EMT and inhibiting epithelial differentiation [65, 66] and the present cytometric analyses of CD44 and ESA expression allow identification of shifts between Epi-CSCs, EMT-CSCs and differentiating cells [31, 32]. Figure 4F is a diagrammatic representation of the altered behaviour of these cell populations observed in response to EGFR blocking. The continuous transit of cells from the self-renewing Epi-CSCs compartment into CD44^low^/ESA^high^ differentiating cells is enhanced by treatment. The increased differentiation of EPI-CSCs, indicated by the increased size of CD44^low^ fractions, decreases the self-renewal of tumour cells and corresponds to a form of “differentiation therapy” [35, 37]. The greater therapeutic sensitivity of the CD44^low^/ESA^high^ cells is likely further to potentiate cell loss in the presence of other therapeutic agents and the combination of EGFR blocking with cisplatin produced such an effect *in vitro*.

Design of synergistic therapy regimes may enable elimination of CSCs and effective monitoring strategies allowing development of such combinations is imperative [11]. The primary aim of this study was to develop methods capable of assessing differential effects of EGFR blocking agents on recently identified CSC subpopulations. Murine transplantation models for studies of CSCs are typically restricted to examination only of patterns of tumour initiation, growth and histological features. Consequently, such *in vivo* models have only limited ability to directly image or identify the therapeutic responses of individual cell subtypes. The present results show that *in vitro* assays allow direct imaging and analysis of individual cell fractions and can therefore accurately detect therapy-relevant shifts in subpopulation compositions. As *in vitro* studies are adaptable to high throughput assays they can also be used to identify differential changes in CSC subpopulations under a wide range of therapeutic conditions. Such assessment of existing and newly-developed drugs may allow rational development of multiagent combinations able to provide overall control of each of the differing plastic CSC subpopulations that generate tumour growth, spread and therapy resistance.

## MATERIALS AND METHODS

### Cell lines, growth conditions and exposure to drugs

The CA1 and Luc4 cell lines, previously isolated from oral squamous cell carcinoma were cultivated as previously described [31, 60] in an epithetial growth medium containing EGF (10 ng/ml) and 10% FBS with 5% CO_2_ at 37°. Cells were plated in triplicate into 96-well plates at 1 × 10^3^ cells per well, cultured overnight and then exposed to Cetuximab (Erbitux, Merck KGaA, Darmstadt, Germany) or Erlotinib (Erlotinib HCL. OSI-744 Cat No. S1023, Selleckchem). Cells were fixed in 4% paraformaldehyde for 15 min, washed in PBS, permeabilized with 0.1% Triton-X for 20 min, and then incubated with Cell Mask Deep Red at a 1:50,000 dilution (Life Technologies) and 1 µg/ml DAPI (Sigma) before image acquisition and analysis. To separately assess drug effects on Epi-EMT and EMT-CSC populations, cells were sorted on the basis of CD44 and ESA staining for investigation by the methods described below.

### Colony and sphere formation assays

To test their clonogenic abilities, cells were trypsinized after 3 days of treatment and 1 × 10^2^ cells in 2 ml of medium added to each well of 6 well plates. After 14 days of growth, cells were fixed in 4% paraformaldehyde, stained with crystal violet (0.04% in 1% ethanol) and colonies measuring at least 2mm in diameter were counted visually. To assess growth in suspension as tumour spheres, 24 well plates were coated with 12 mg/ml PolyHEMA (2-hydroxyethyl methacrylate, Sigma) in 95% ethanol to prevent attachment, and then seeded 1 × 10^3^ treated cells/well in 0.5 ml medium containing 1% methylcellulose. After two weeks, the number of tumour spheres larger than 300 μM were counted.

### Flow cytometry

For fluorescent activated cell sorting (FACS), cells were detached from cultures using trypsin-EDTA at 37° C (Life Technologies). Cells were stained with anti-CD44 antibody (anti-CD44-FITC, clone G44–26, BD Biosciences) and anti-epithelial specific antigen (anti-ESA-APC, clone HEA-125, Miltenyi Biotec) both at 1:100 in PBS for 15 min in the dark. The DAPI nuclear dye (Sigma) was used at 200 ng/ml to exclude dead cells. For each flow cytometry assay, 100,000 cells were plated and 30,000 live cells counted on a Canto Cytometer (BD Biosciences) and analyzed using FACS Diva software (version 6.1.1, BD Biosciences). Using a FACSAria III (Becton Dickenson), cells were also fractionated (Biddle *et al.*, 2011) as (a) CD44^high^/ESA^low^ cells (EMT-CSC phenotype), (b) the top 5% of CD44 expressing cells that also showed ESA expression (Epithelial CSC phenotype - EpiCSC), and (c) the 5% of cells with the lowest CD44 expression also expressing ESA (differentiating cells).

### Proliferation assays

Total populations of the CA1 and Luc4 cell lines, together with their CD44^high^/ESA^low^, CD44^high^/ESA^high^, and CD44^low^ fractions were plated in triplicate at 1 × 10^3^ cells per well into 96-well plates. 24 hours later cells were treated with a range of concentrations of Cetuximab or Erlotinib for 1, 2 or 3 days. Cells were then fixed and stained with Cell Mask Deep Red and DAPI and assayed using the GE InCell 1000 imaging system. Based on the results of these assays, for subsequent experiments cells were exposed to Cetuximab at 100 µg/ml and at 500 ng/ml. To assess effects on cell number of EGFR blocking in the presence of Cisplatin, cells were treated with Cetuximab or Erlotinib together with Cisplatin.

After 1, 2 and 3 days of treatment, 2-Deoxy-5-Iodouridine (IdU) was added to the culture medium for 2 hours at a final concentration of 50 µM to label cells in the S phase of the cell cycle. Cells were then washed in PBS before fixing with 4% paraformaldehyde for 15 min, re-washing and permeabilized with blocking solution (0.5% BSA, 0.2% Triton in PBS) for 30 min at room temperature. Cells were rewashed and incubated for 1 hour at RT with DNase (0.5 U/µl), 3 mM MgCl_2_ and anti-IdU/BrdU antibody (BD Address Cat# 347580) diluted to 100 µl/ml in blocking solution. After washing 3 times in PBS, cells were incubated in PBS containing an anti-mouse-488 secondary antibody (1:500) (Alexafluor, Life Tech), Cell Mask Deep Red and DAPI for 1 hour in the dark at RT.

### Apoptosis assays

Cell lines and sub-factions were treated with Cetuximab and Erlotinib before double staining with Annexin V-FITC and DAPI, or staining with anti-ESA-APC (Miltenyi Biotec), anti-CD44-PE (BD Pharmingen), Annexin V-FITC (BD Pharmingen) and DAPI. Samples were examined on a Canto Cytometer (BD Bioscience) and analyzed with the FACS Diva version 6.1.1 (BD Biosciences) software.

### RNA extraction, cDNA synthesis and QPCR

RNA was extracted using the RNeasy^®^ micro kit (Qiagen, Netherlands) followed by reverse transcription using the Superscript III first strand synthesis supermix (Invitrogen, USA). RT qPCR was performed in an ABI 7500 real-time PCR system (Applied Biosystems, USA) using Power SYBR^®^ green mix (Applied Biosystems, USA). QPCR cycling conditions were: 95°C for 10 mins, [95° C for 15 seconds, 60° C for 30 seconds, 72° C for 40 seconds] (40 cycles), 95° C for 60 seconds, followed by dissociation curve analysis. Reverse transcribed Human Total Reference RNA (Stratagene) was used to generate a standard curve. Primer sequences were, designed using Primer-BLAST (NCBI).

### Protein extraction and western blotting

Cells were grown to 70% confluence before being harvested and treated with 220 µl of pre-heated protein lysis buffer consisting of 1 mM Tris-Hcl, 10% (v/v) ammonium persulphate (APS), 0.5 M sodium orthovanadate (Sigma) at pH 8.4. Cells were removed and heated at 95° C for 2 minutes. Protein was quantified (DC Protein Assay, Bio-Rad) and 50 µl of 5x protein loading dye (Fisher Scientific) was added to the remaining 200 µl of sample. The primary antibodies with a standard western blotting technique were phospho-ERK (9106S, Cell Signaling), phospho-EGFR (04-339, Millipore), PE-EGFR (555997, BD Biosciences) and β-Actin (sc47778, Santa Cruz). Secondary IgG-conjugated horse-radish peroxidase Abs (Dako) were followed by ECL detection (GE Healthcare).

### Immunofluorescence

Cells were treated and fixed as above before being permeabilized with 0.1% Triton-X for 20 min, and then incubated with either Vimentin (DAKO 1:500) or E-Cadherin (Abcam 1:100). Wells were then washed 3 times in PBS, before incubated in PBS containing an anti-mouse-488 secondary antibody (Alexafluor, Life Tech 1:500), Cell Mask Deep Red at a 1:50,000 dilution (Life Technologies) and 1 µg/ml DAPI (Sigma).

### Labeling of cell transitions and cell motility

To examine directly the transitions occurring between cells of the EMT-CSC and the EPI-CSC compartments, cell lines were transduced with a constitutively expressed GFP plasmid. EMT-GFP and Epithelial Non-GFP cells were then sorted out from their parental populations and combined before replating and exposing to either Cetuximab or Erlotinib. Scratch assays were used to assess enhanced migration associated with a shift to EMT. Cells were plated for 24 hours, scratched, and then washed with PBS before replacing medium with or without the EGFR inhibitors. Cells were then imaged at 0, 16 and 24 hours and the degree of closure of the scratches analysed as outlined below.

### Imaging and image analysis

Images were taken on an IN Cell 1000 Analyser (GE Healthcare) automated system. Images were processed and analyzed using GE IN Cell Developer software (Version 1.9.1).

### Statistics

All assays were independently repeated a minimum of 3 times and significance of differences calculated using paired *t*-tests and indicated in the Figures as: *P* value <0.05 = ^*^, <0.01 = ^**^ or <0.001 = ^***^. Error bars represent the standard error of the mean (SEM).
